# Association of type 2 diabetes with left atrioventricular coupling and myocardial deformation in hypertension: a 3.0 T cardiac magnetic resonance study

**DOI:** 10.3389/fcvm.2026.1753368

**Published:** 2026-02-26

**Authors:** Zhi-ming Li, Xuan Li, Xue-chun Guan, Ying-yue Chen, Feng-qiu Ruan, Li-ling Long

**Affiliations:** Department of Radiology, The First Afﬁliated Hospital of Guangxi Medical University, Nanning, Guangxi, China

**Keywords:** cardiac magnetic resonance, hypertension, left atrial dysfunction, left atrioventricular coupling index, type 2 diabetes mellitus

## Abstract

**Background:**

Hypertension (HTN) and type 2 diabetes mellitus (T2DM) frequently coexist, synergistically increasing heart failure risk. The specific incremental impairment of T2DM on left atrioventricular mechanics in hypertensive patients remains poorly characterised. This study aimed to assess whether the presence of T2DM is associated with further alterations in cardiac deformation and atrioventricular coupling beyond hypertension alone.

**Methods:**

We performed a retrospective analysis including 130 hypertensive patients (74 HTN-only, 56 HTN-T2DM) and 42 age- and sex-matched controls, all undergoing 3.0 T cardiac magnetic resonance. Intergroup comparisons of atrioventricular function and deformation were adjusted for age, sex, BMI, heart rate and SBP using ANCOVA. Multivariable regression was applied to identify independent determinants of left atrioventricular deformation and coupling, and the independent effect of T2DM.

**Results:**

Key cardiac parameters demonstrated graded impairment from controls to HTN-only and HTN-T2DM groups (all *P* < 0.05). This progressive decline was evident in LV systolic function [peak global longitudinal strain: −19.99% (−21.16, −19.13) vs. −17.15% (−19.18, −15.58) vs. −16.03% (−17.86, −13.94)] and LA phasic function [reservoir strain/εs: 48 ± 10% vs. 40 ± 14% vs. 33 ± 15%; conduit strain/εe: 33% (27, 36) vs. 21% (16, 31) vs. 16% (10, 24)]. Consequently, the left atrioventricular coupling index (LACI) was significantly elevated in the HTN-T2DM group [24% (23, 30)] compared to both the HTN-only [22% (18, 28)] and control groups [17% (16, 20)]. Multivariable linear regression analysis indicated that in the overall population, hypertensive patients with and without T2DM independently reduced left atrial εs, εe and left ventricular GLS, and significantly increased LACI; the detrimental effects were more marked in the HTN-T2DM group (all *P* < 0.05). In the hypertensive subgroup, after adjusting for confounding factors, comorbid T2DM remained an independent risk factor for reduced LA reservoir function (εs: *β* = −6.09, *P* = 0.018), impaired LA conduit function (εe: *β* = −5.58, *P* = 0.002), and worsened LV systolic function (GLS: *β* = −1.37, *P* = 0.010).

**Conclusion:**

Hypertensive patients with T2DM demonstrate more significant impairment of myocardial deformation, and worse left atrioventricular uncoupling compared with those with HTN alone, underscoring the need for integrated cardiometabolic management in this high-risk population.

## 1 Introduction

Hypertension (HTN) and type 2 diabetes mellitus (T2DM) are major public health concerns that frequently coexist. Individuals with hypertension face a 2.5-fold increased risk of developing T2DM (1, 2). This comorbidity synergistically amplifies cardiovascular risk through dual pathways of hemodynamic stress and metabolic dysregulation, culminating in a substantially elevated risk of heart failure and associated mortality (3).

The left atrium and ventricle constitute an integrated functional unit. The LA contributes not only through endocrine and regulatory functions but also by mechanically governing LV filling and cardiac output (4, 5). This relationship is bidirectional, as LA phasic function is itself dependent on LV performance. This interdependence creates a vicious cycle whereby dysfunction in one chamber exacerbates impairment in the other (6). Therefore, assessing their coupled interaction, rather than isolated parameters, provides a more comprehensive reflection of atrioventricular health and a superior approach for predicting cardiovascular risk.

While the independent detrimental effects of HTN or T2DM on left ventricular function are well-established (7–9), the specific additional impact of T2DM on the integrated left atrioventricular system, particularly regarding myocardial deformation and coupling in hypertensive patients, remains poorly understood (10, 11).

Cardiac magnetic resonance (CMR) provides a comprehensive and reproducible evaluation of cardiac structure and function, including the detection of subtle changes in left atrioventricular coupling (12). In addition, CMR feature-tracking (CMR-FT) strain analysis allows for precise quantification of myocardial deformation (13). These technical capabilities provide a powerful approach for identifying subclinical atrioventricular dysfunction in individuals with hypertension.

Therefore, this study aimed to investigate subclinical cardiac dysfunction and atrioventricular coupling impairment in hypertensive patients using cardiac magnetic resonance, and to explore whether the presence of T2DM is associated with incremental alterations in these cardiac functional and atrioventricular coupling profiles.

## 2 Methods

### 2.1 Study population

**Figure 1:**
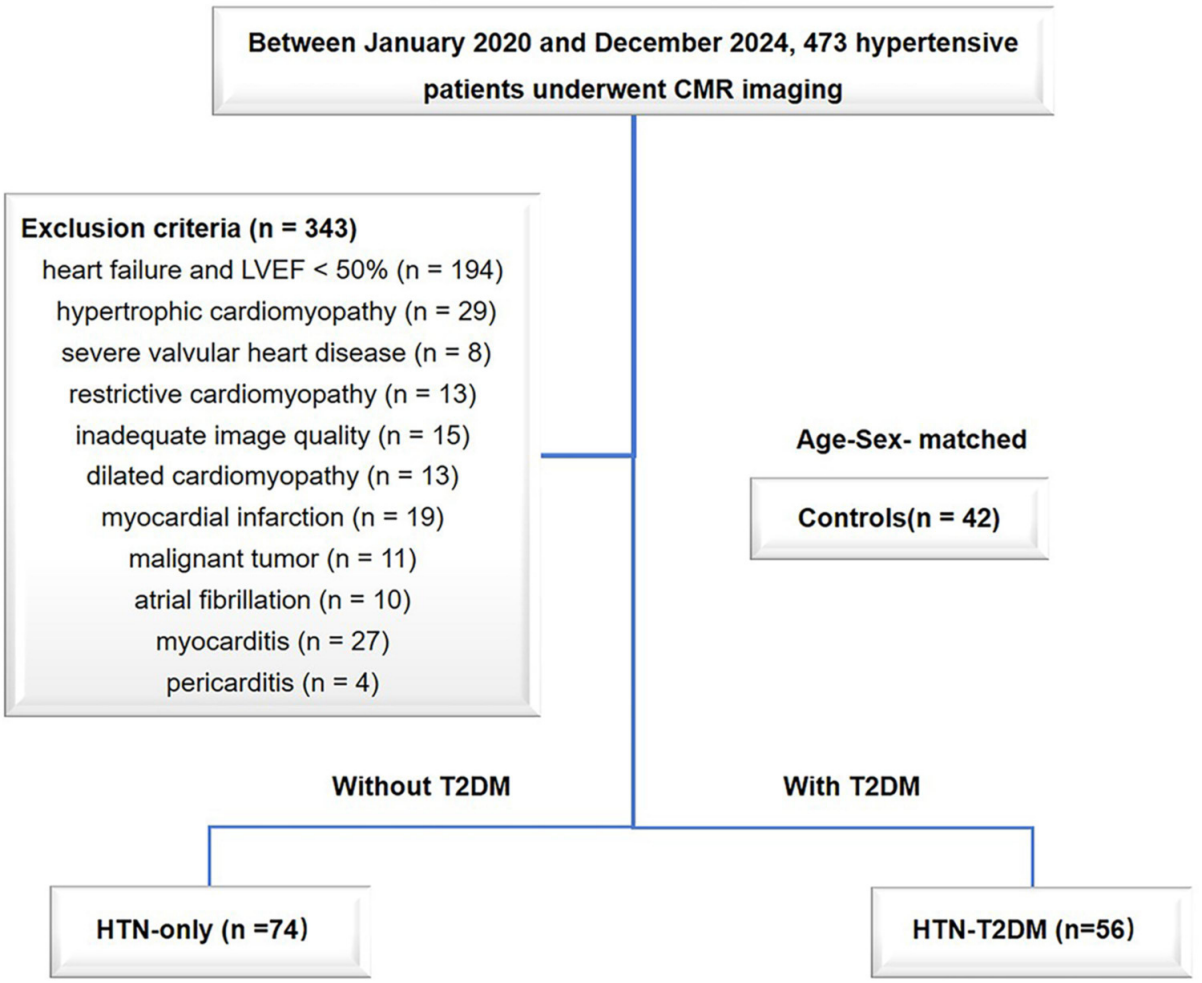
Flow diagram of the study patients. CMR, cardiac magnetic resonance; HTN, hypertension; T2DM, type 2 diabetes mellitus; LVEF, left ventricular ejection fraction.

A total of 473 hypertensive patients were enrolled between January 2020 and December 2024, all of whom underwent CMR examinations. Hypertension was defined as a systolic blood pressure (SBP) > 140 mmHg and/or diastolic blood pressure (DBP) > 90 mmHg based on at least two office readings (14). The exclusion criteria were as follows (Figure 1): heart failure with left ventricular ejection fraction (LVEF) < 50% (*n* = 194), hypertrophic cardiomyopathy (*n* = 29), restrictive cardiomyopathy (*n* = 13), dilated cardiomyopathy (*n* = 13), severe valvular heart disease (*n* = 8), myocardial infarction (*n* = 19), myocarditis (*n* = 27), malignant tumors (*n* = 11), pericarditis (*n* = 4), atrial fibrillation (*n* = 10) and inadequate image quality (*n* = 15). Finally, 130 HTN patients were included in the study and divided into two groups on the basis of the absence or presence of T2DM: HTN-only (*n* = 74) and HTN-T2DM (*n* = 56). The diagnosis of T2DM was based on the current American Diabetes Association guideline recommendations (15). The control group included 42 age- and sex-matched individuals who underwent CMR for the evaluation of non-specific symptoms (e.g., chest pain or tightness) and were found to have entirely normal cardiac structure and function.

### 2.2 Clinical data collection

Patient clinical data were collected from medical records. The following covariates were included in the analysis: age, sex, body mass index (BMI), heart rate (derived from the ECG recorded during the CMR scan), systolic and diastolic blood pressure (SBP/DBP, retrieved from routine clinical records closest to the date of the CMR examination), hypertension classification, duration of hypertension or diabetes, glycated haemoglobin A1c (HbA1c), fasting blood glucose (FBG), total cholesterol (TC), triglycerides (TG), high-density lipoprotein cholesterol (HDL-C), and low-density lipoprotein cholesterol (LDL-C). The triglyceride‒glucose (TyG) index serves as a biomarker of insulin resistance and predicts heart failure risk. It is calculated as (16):

$$TyG=\ln\left[\frac{\left(TG\left(\frac{\mathrm{mg}}{\mathrm{dL}}\right)*\mathrm{FBG}\;\left(\frac{\mathrm{mg}}{\mathrm{dL}}\right)\right)}{2}\right]$$

### 2.3 CMR protocol

All CMR examinations were performed via a 3.0 Tesla scanner (MAGNETOM Prisma, Siemens Healthineers, Erlangen, Germany) equipped with an 18-channel body phased-array coil. Participants were positioned supine, and ECG-gated acquisitions were performed during breath‒hold intervals to minimize motion artifacts. Cine imaging was acquired via a balanced steady-state free precession sequence with the following parameters: TR/TE = 40.35/1.22 ms; slice thickness = 8.0 mm (gap: 2 mm); FOV = 250 × 300 mm^2^; matrix = 208 × 139; flip angle = 40°. The protocol comprised cine imaging in short-axis, 2-, 3-, and 4-chamber views.

### 2.4 CMR analysis

#### 2.4.1 LA and LV volumetric function analysis

The quantitative analysis of CMR images was performed using cvi42 software (version 5.16.0; Circle Cardiovascular Imaging, Calgary, Alberta, Canada) with computer-assisted manual planimetry. The biplane area-length method was employed to automatically calculate LA volumes at three phases (17). The maximum LA volume (LAVmax) was measured at the end of LV systole, the preatrial contraction LA volume (LAVpre) was measured before the onset of atrial contraction, and the LAVmin was measured at the end of LV diastole. The LA appendage and pulmonary veins were excluded from the measurement of LA volume. Phasic LAV was normalised to body surface area (BSA), resulting in the LAVmax index (LAVmaxi), LAVpre index (LAVprei), and LAVmin index (LAVmini). LA ejection fractions were calculated using the following formulas (10):

LA total ejection fraction (LA-TEF):

$$\mathrm{LA}-\mathrm{TEF}=\frac{\mathrm{LA}V_{\mathrm{Max}}-\;\mathrm{LA}V_{\mathrm{Min}}}{\mathrm{LA}V_{\mathrm{Max}}}*100$$

LA passive ejection fraction (LA-PEF):

$$\mathrm{LA}-\mathrm{PEF}=\frac{\mathrm{LA}V_{\mathrm{Max}}-\;\mathrm{LA}V_{\mathrm{Pre}}}{\mathrm{LA}V_{\mathrm{Max}}}*100$$

LA active ejection fraction (LA-AEF):

$$\mathrm{LA}-\mathrm{AEF}=\frac{\mathrm{LA}V_{\mathrm{Pre}}-\;\mathrm{LA}V_{\mathrm{Min}}}{\mathrm{LA}V_{\mathrm{Pre}}}*100$$

The endocardial and epicardial borders at LV end-systole and end-diastole on the two-chamber short-axis images were automatically delineated and manually corrected to calculate the LV volumetric function parameters. These parameters included the LVEDV, LV end-systolic volume (ESV), LV stroke volume (SV), LVEF, LV mass (LVM), cardiac output (CO), and cardiac index (CI). Papillary muscles were included within the LV volume. LVMi, LVEDVi, and LVESVi were standardized by BSA. Additionally, the LV remodelling index (LVRi), measured as the LV mass/LVEDV, was included for analysis. The left atrioventricular coupling index (LACI) was determined by computing the ratio of the LAVmin to the LVEDV (18).

#### 2.4.2 LA and LV feature tracking analysis

**Figure 2:**
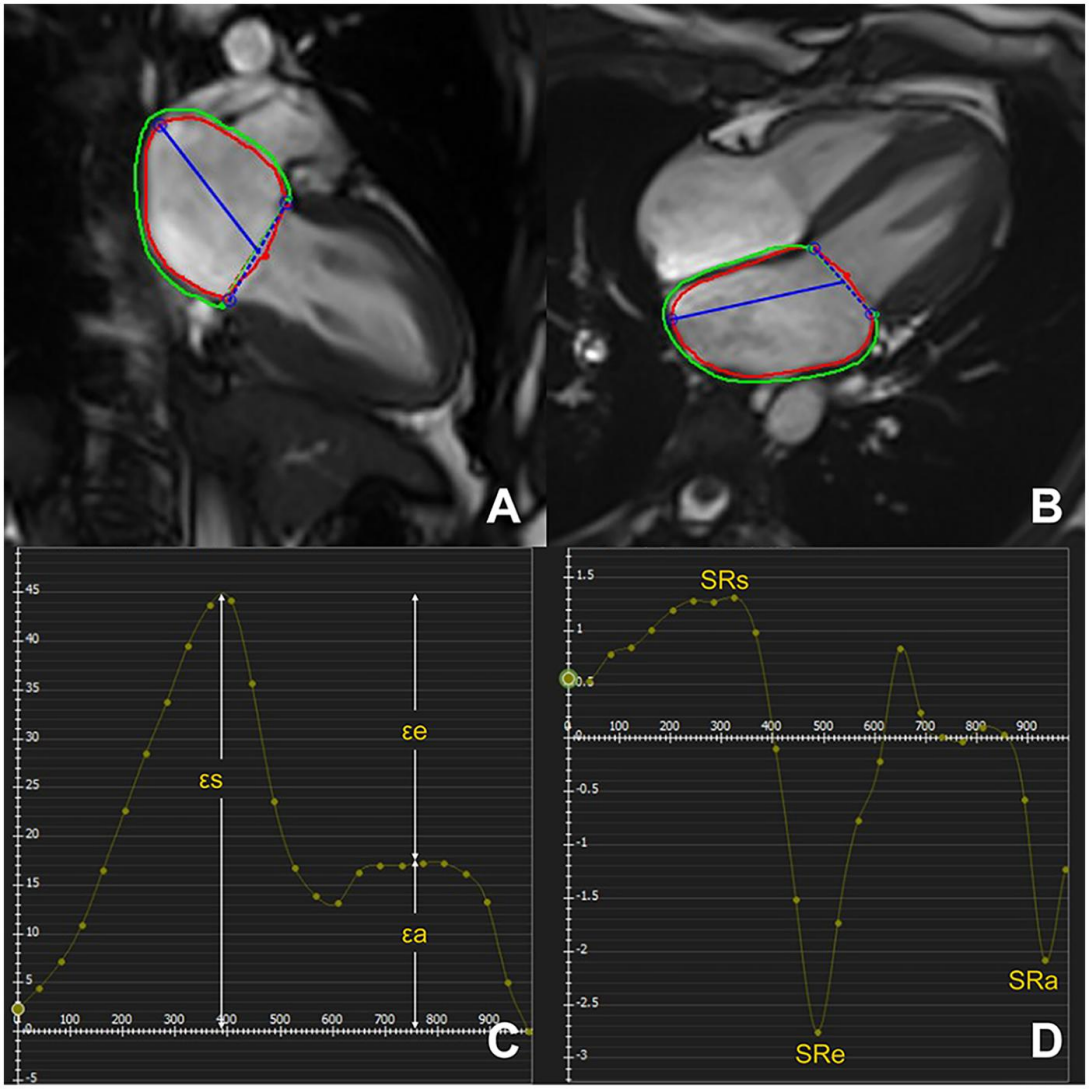
Left atrial strain quantification via CMR feature tracking in controls. LA longitudinal strain in the two- and four-chamber views at end-diastole **(A,B)**. LA strain and strain rate curves **(C,D**). εs, total strain; εe, passive strain; εa, active strain; SRs, peak positive strain rates; SRe, peak early negative strain rates; SRa, peak late negative strain rates.

The tissue tracking module of cvi42 was utilized to analyse LA myocardial strains. The epicardial and endocardial borders of the LA were manually delineated via a point‒click approach in the apical two-chamber and four-chamber views (Figure 2). The LA appendage and pulmonary veins were also excluded. An automated tracking algorithm was subsequently applied to propagate and delineate the atrial borders in the remaining slices. To ensure the accuracy of the strain parameters, an experienced cardiac MR radiologist reviewed the tracking performance. Any inaccuracies in the automated tracking were manually adjusted to achieve precise delineation. Myocardial strain parameters comprised (19): LA reservoir strain (εs), passive strain (εe), active strain (εa), peak positive strain rate (SRs), peak early negative strain rate (SRe), and peak late negative strain rate (SRa). εs/SRs represented LA reservoir function, εe/SRe represented LA conduit function, and εa/SRa represented LA pump function.

The 3D tissue tracking module was used to analyse the LV myocardial strain by loading long-axis two-chamber, four-chamber, and short-axis slices. Finally, global strain variables, including peak global longitudinal strain (GLS), circumferential strain (GCS), and radial strain (GRS), peak systolic strain rate (PSSR), and peak diastolic strain rate (PDSR) in the radial, circumferential, and longitudinal directions, were automatically computed (19).

### 2.5 Reproducibility

The CMR images of 30 HTN patients and 15 controls were randomly selected to assess interobserver variability (ZL vs. YC). The intraobserver measurements were performed by Dr. ZL in a random order, with an interval of at least one month between assessments, to minimize recall bias. Two radiologists were blinded to the clinical information of the patients with HTN.

### 2.6 Statistical analysis

The normality of continuous variables was assessed via the Shapiro‒Wilk test. Accordingly, normally distributed continuous variables are presented as the mean ± standard deviation and non-normally distributed variables as the median (interquartile range, IQR). Categorical variables are expressed as frequencies (percentages). One-way analysis of variance (ANOVA) or the Kruskal‒Wallis rank sum test was used to compare the baseline characteristics among groups, and Tukey's HSD test or Dunn's test with Holm correction was applied for *post-hoc* multiple comparisons when the overall test was significant. The chi-square test was used to analyse differences in categorical variables. Groupwise comparisons of CMR-derived parameters were performed by analysis of covariance (ANCOVA), adjusted for age, sex, BMI, heart rate, and SBP, with multiple comparisons corrected using the Holm-Bonferroni method. Spearman's rank correlation (ρ) was performed across the entire cohort to examine associations between key metabolic indices (diabetes duration, HbA1c) and cardiac function, as well as between LACI and left atrial/ventricular strain parameters. Multivariable regression analyses were performed in the overall population to identify independent determinants of atrial and ventricular function and atrioventricular coupling status after covariate adjustment, and to assess the independent effect of study group. Analyses were also conducted in hypertensive patients alone to determine independent predictors of these cardiac parameters, focusing on comorbid diabetes and metabolic profiles. Collinearity diagnostics confirmed no severe multicollinearity among variables, with all variance inflation factors (VIF) < 3. Intraclass correlation coefficients [ICCs; two-way mixed-effects model, ICC (3,1)] were calculated to evaluate interobserver and intraobserver agreement. A two-sided *P* value of <0.05 was considered statistically significant. All the statistical analyses were performed via R software, version 4.5.1.

## 3 Results

### 3.1 Baseline characteristics of the study population

Table 1 summarizes the baseline clinical and metabolic characteristics of the study participants. No significant differences were observed in age, sex distribution, BMI, BSA, or heart rate among the three groups. Both the HTN-only and HTN-T2DM groups had significantly higher SBP and DBP compared with the control group (all *P* < 0.001). Among patients with hypertension, the majority were classified as stage II or III, with a median hypertension duration of 5–6 years. With regard to metabolic profiles, the HTN-T2DM group showed significantly worse glycemic control, with higher HbA1c and FBG levels compared to both the control and HTN-only groups (all *P* < 0.001). They also exhibited higher TyG index [median 8.87 (IQR 8.44, 9.31)] than both the control [8.39 (7.97, 8.80); adjusted *P* *=* 0.0007] and HTN-only groups [8.60 (8.26, 9.02); adjusted *P* *=* 0.047]. In terms of lipid profiles, with the HTN-T2DM group exhibiting significantly higher TG and lower HDL-C levels compared to the control group (adjusted *P* = 0.036 and *P* = 0.0006, respectively). TC and LDL-C levels did not differ significantly among the groups. In the HTN-only and HTN-T2DM groups, the usage rates of antihypertensive drugs were as follows: ACEI/ARB (14.9% vs. 19.6%), ARNI (9.5% vs. 10.7%), β-blockers (36.5% vs. 33.9%), CCB (36.5% vs. 42.9%), and diuretics (9.5% vs. 7.1%). Statins were used for lipid-lowering in 20.3% of HTN-only patients and 30.4% of HTN-T2DM patients. Hypoglycemic medications were only administered in the HTN-T2DM group, with metformin (30.4%), SGLT2i (23.2%), insulin (16.1%), *α*-glucosidase inhibitors (10.7%) and sulfonylureas (10.7%) being the main agents.

**Table 1:** Baseline demographic and clinical features of the study cohort.

Characteristics	Controls *n* = 42	HTN-only *n* = 74	HTN-T2DM *n* = 56
Demographics			
Age, years	50 ± 9	53 ± 14	54 ± 8
Sex/male, *n* (%)	23 (54.8%)	42 (56.8%)	33 (58.9%)
BMI, kg/m^2^	24.0 (22.6, 26.8)	24.9 (22.4, 27.6)	25.8 (23.4, 28.3)
BSA, kg/m^2^	1.75 ± 0.20	1.77 ± 0.23	1.82 ± 0.20
Heart rate, bpm	79 ± 14	77 ± 14	75 ± 13
SBP, mmHg	118 (110, 127)	136 (120, 152)*	134 (119, 151)*
DBP, mmHg	73 (66, 78)	81 (71, 92)*	79 (73, 85)*
HTN classification			
Ⅰ		16 (21.6%)	8 (14.3%)
Ⅱ		26 (35.1%)	20 (35.7%)
Ⅲ		32 (43.2%)	28 (50.0%)
HTN duration, years		5.0 (3.0, 9.0)	6.0 (3.0, 8.0)
T2DM duration, years			7.0 (3.0, 10.5)
Laboratory data			
HbA1c (%)	5.40 (5.20, 5.80)	5.80 (5.40, 6.10)	7.20 (6.20, 8.25)*^,♯^
FBG, mmol/L	4.71 (4.41, 5.12)	4.73 (4.37, 5.10)	5.62 (4.65, 7.42)*^,♯^
TC, mmol/L	4.45 (3.97, 4.92)	4.26 (3.45, 5.17)	4.16 (3.58, 4.73)
TG, mmol/L	1.09 (0.77, 1.71)	1.39 (1.07, 2.01)	1.43 (1.14, 2.12)*
HDL-C, mmol/L	1.25 (1.03, 1.37)	1.12 (0.90, 1.26)	1.02 (0.86, 1.15)*
LDL-C, mmol/L	2.61 ± 0.72	2.44 ± 0.97	2.40 ± 0.77
TyG	8.39 (7.97, 8.80)	8.60 (8.26, 9.02)	8.87 (8.44, 9.31)*^,♯^
Treatment			
ACEI/ARB		11 (14.9%)	11 (19.6%)
ARNI		7 (9.5%)	6 (10.7%)
β-blockers		27 (36.5%)	19 (33.9%)
CCB		27 (36.5%)	24 (42.9%)
Diuretics		7 (9.5%)	4 (7.1%)
Statins		15 (20.3%)	17 (30.4%)
Metformin			17 (30.4%)
α–glucosidase Inhibitors			6 (10.7%)
Sulfonylureas			6 (10.7%)
SGLT2i			13 (23.2%)
Insulin			9 (16.1%)

Data are presented as the Median (IQR); *n* (%); Mean ± SD.

*P* values are corrected from Tukey's HSD or Holm-corrected Dunn's test.

BMI, body mass index; BSA, body surface area; SBP, systolic blood pressure; DBP, diastolic blood pressure; HbA1c, glycated hemoglobin A1c; FBG, fasting blood glucose; TC, total cholesterol; TG, triglyceride; HDL-C, high-density lipoprotein cholesterol; LDL-C, low-density lipoprotein cholesterol; TyG, triglyceride-glucose index; ACEI, angiotensin-converting enzyme inhibitor; ARB, angiotensin II receptor blocker; ARNI, angiotensin receptor-neprilysin inhibitor; CCB, calcium channel blocker; SGLT2i, sodium-glucose cotransporter-2 inhibitor.

* *P* < 0.05 vs. controls.

♯ *P* < 0.05 vs. HTN-only group.

### 3.2 Comparisons of CMR-derived LA and LV volumetric parameters

After adjustment for age, sex, BMI, heart rate, and SBP, conventional LV volumetric and functional parameters including EDVi, ESVi, SVi, CI, and CO showed no significant differences among the three groups (all overall ANCOVA *P* > 0.05). However, both hypertensive subgroups exhibited significantly higher LVMi and LVRi compared with the control group (both *P* < 0.001). *post-hoc* comparisons revealed no significant difference in LVMi between the two hypertensive groups (*P* = 0.3632), while LVRi showed a trend toward further elevation in the HTN-T2DM group (*P* = 0.0938).

**Figure 3:**
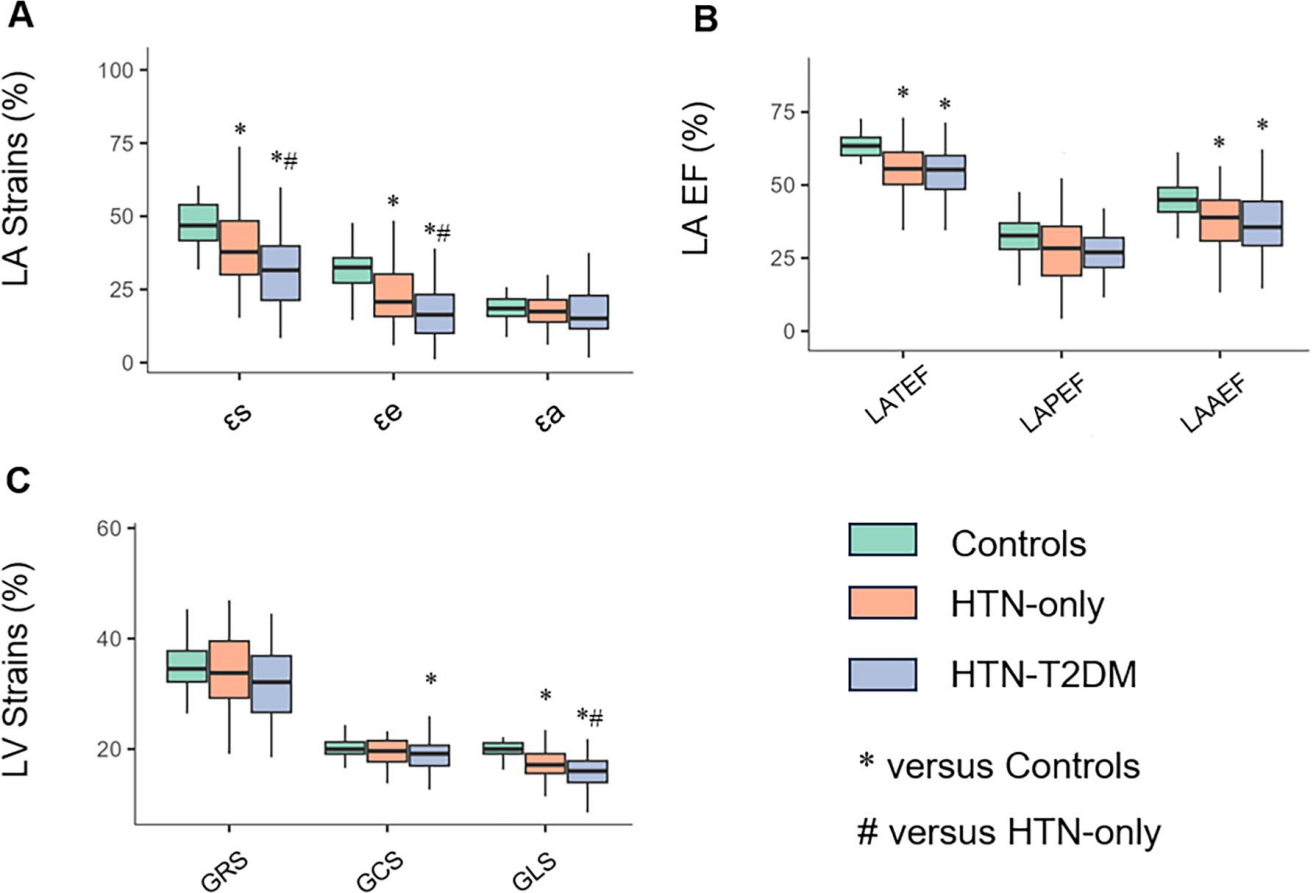
Differences in left atrioventricular functional parameters among control, HTN-only, and HTN-T2DM groups. **(A)** Left atrial reservoir (εs), conduit (εe) and pump (εa) function. **(B)** Left atrial phasic ejection fractions, including total (LA-TEF), passive (LA-PEF), and active (LA-AEF) emptying fractions. **(C)** LV peak global strain in the radial (GRS), circumferential (GCS), and longitudinal (GLS) directions (absolute values change are displayed). εs, total strain; εe, passive strain; εa, active strain; LA, left atrial; TEF, total emptying fraction; PEF, passive emptying fraction; AEF, active emptying fraction; GRS, global radial strain; GCS, global circumferential strain; GLS, global longitudinal strain. *, *P* < 0.05 vs. controls; ♯, *P* < 0.05 vs. HTN-only group.

Regarding LA phasic function, both hypertensive groups had significantly lower LA-TEF and LA-AEF than the controls (all *P* < 0.001, Figure 3). LA-PEF did not reach statistical significance (overall *P* = 0.0589). LA volume indices demonstrated selective alterations: LAVmini and LAVprei were significantly elevated in hypertensive groups compared to controls (overall *P* < 0.01), while LAVmaxi showed borderline significance (overall *P* = 0.0669).

**Figure 4:**
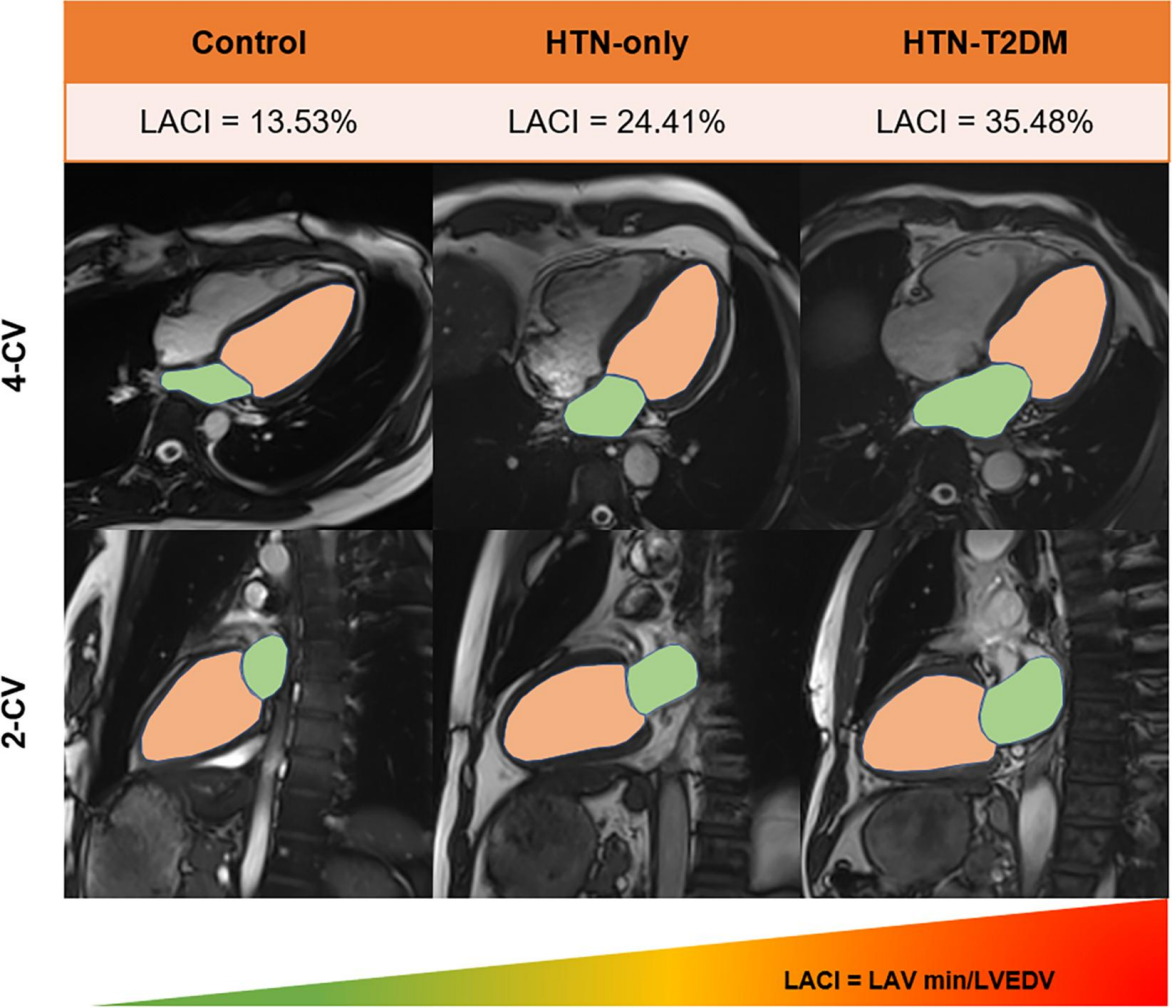
Comparison of LACI variations in different groups. Schematic illustration shows the method for assessing LACI by CMR. LACI indicates volume ratio differences between LAV min (green) and LVEDV (yellow) in 2/4-chamber views for control, HTN-only and HTN-T2DM groups respectively. LAV min, the minimum volume of left atrium; LVEDV, end-diastolic volume of left ventricle; LACI, left atrioventricular coupling index.

Left atrioventricular coupling assessed by LACI was significantly impaired in both hypertensive subgroups vs. controls (overall *P* < 0.001). Notably, LACI was further elevated in the HTN-T2DM group compared with the HTN-only group (*P* = 0.0445), suggesting additional deterioration with superimposed diabetes (Figure 4). The details are shown in Table 2.

**Table 2:** Comparisons of left atrial and ventricular volume parameters among groups after adjustment for age, sex, BMI, heart rate, and SBP.

Characteristics	Controls *n* = 42	HTN-only *n* = 74	HTN-T2DM *n* = 56
LV volumetric function			
EF (%)	61 (59, 66)	64 (57, 69)	64 (56, 69)
CO, L/min	5.66 (4.64, 6.65)	5.73 (5.03, 6.76)	5.91 (5.03, 6.90)
CI, L/min/m^2^	3.26 (2.82, 3.79)	3.26 (2.76, 3.72)	3.21 (2.74, 3.65)
EDVi, mL/m^2^	75 (67, 83)	82 (68, 90)	78 (68, 86)
ESVi, mL/m^2^	29 (25, 33)	28 (22, 34)	28 (23, 36)
SVi, mL/m^2^	47 ± 8	50 ± 10	48 ± 11
Mi, g/m^2^	42 (38, 48)	58 (50, 68)*	60 (49, 78)*
Ri, g/mL	0.56 (0.52, 0.63)	0.76 (0.63, 0.90)*	0.81 (0.68, 0.97)*
LA volumetric function			
TEF (%)	63 (60, 66)	56 (50, 61)*	55 (48, 60)*
PEF (%)	32 ± 7	28 ± 10	27 ± 8
AEF (%)	45 ± 7	38 ± 10*	36 ± 11*
MINi, ml/m^2^	12 (10, 14)	16 (12, 21)*	16 (12, 23)*
MAXi, ml/m^2^	34 (28, 39)	38 (30, 46)	37 (30, 49)
Prei, ml/m^2^	22 (19, 26)	27 (20, 34)*	25 (21, 37)*
LACI (%)	17 (16, 20)	22 (18, 28)*	24 (23, 30)*^,♯^

Data are presented as the Median (IQR); *n* (%); Mean ± SD.

*P*-values were corrected for multiple comparisons using the Holm-Bonferroni procedure.

EF, ejection fraction; CO, cardiac output; CI, cardiac index; EDVi, end diastolic volume index; ESVi, end systolic volume index; SVi, stroke volume index; Mi, myocardial index; Ri, remodeling index; TEF, total ejection fraction; PEF, passive ejection fraction; AEF, active ejection fraction; MINi, minimum index; MAXi, maximum index; Prei, preatrial contraction index; LACI, left atrioventricular coupling index.

* *P* < 0.05 versus controls.

^♯^ *P* < 0.05 versus HTN-only group.

### 3.3 Comparisons of CMR-derived LA and LV strain parameters

Table 3 summarizes alterations in cardiac deformation parameters. After adjustment for covariates, LV strain analysis revealed a graded deterioration across groups. The HTN-T2DM group demonstrated significantly reduced GLS (*P* < 0.0001), GCS (*P* = 0.0035), and PDSR.l (*P* < 0.0001) compared to controls, with GRS showing borderline reduction (*P* = 0.0201). PDSR.r and PDSR.c were also lower in the HTN-T2DM group vs. controls (*P* = 0.0295 and *P* = 0.0159, respectively). In the HTN-only group, only GLS and PDSR.l were significantly impaired relative to controls (both *P* < 0.0001), with PDSR.c showing a trend toward reduction (*P* = 0.0665). Notably, GLS was further reduced in the HTN-T2DM group compared with the HTN-only group (*P* = 0.0017).

**Table 3:** Comparisons of left atrial and ventricular strain parameters among groups after adjustment for age, sex, BMI, heart rate, and SBP.

Characteristics	Controls *n* = 42	HTN-only *n* = 74	HTN-T2DM *n* = 56
LV strain parameters			
GRS (%)	35 ± 6	34 ± 7	32 ± 7*
GCS (%)	−19.98 (−19.12, −21.30)	−19.65 (−17.68, −21.56)	−19.16 (−16.84, −20.70)*
GLS (%)	−19.99 (−19.13, −21.16)	−17.15 (−15.58, −19.18)*	−16.03 (−13.94, −17.86)*^,♯^
PSSR-r (s^−1^)	1.62 (1.42, 1.87)	1.71 (1.47, 2.08)	1.54 (1.36, 1.96)
PSSR-c (s^−1^)	1.00 (0.88, 1.12)	0.97 (0.87, 1.11)	0.96 (0.87, 1.13)
PSSR-l (s^−1^)	0.99 (0.85, 1.10)	0.91 (0.77, 1.05)	0.91 (0.75, 1.09)
PDSR-r (s^−1^)	1.83 (1.44, 2.29)	1.74 (1.30, 2.09)	1.46 (1.16, 1.85)*^,♯^
PDSR-c (s^−1^)	0.97 (0.82, 1.13)	0.88 (0.72, 0.93)	0.78 (0.63, 0.92)*
PDSR-l (s^−1^)	1.05 (0.93, 1.20)	0.85 (0.67, 1.00)*	0.82 (0.66, 0.99)*
LA strain parameters			
εs (%)	48 ± 10	40 ± 14*	33 ± 15*^,♯^
εe (%)	33 (27, 36)	21 (16, 31)*	16 (10, 24)*^,♯^
εa (%)	18 ± 4	18 ± 6	17 ± 9
SRs (s^−1^)	1.98 (1.60, 2.20)	1.68 (1.30, 2.20)*	1.35 (1.10, 1.72)*^,♯^
SRe (s^−1^)	2.90 (2.40, 3.55)	1.82 (1.20, 2.70)*	1.64 (0.95, 1.99)*^,♯^
SRa (s^−1^)	2.40 ± 0.67	2.10 ± 0.80	1.87 ± 0.93

Data are presented as the Median (IQR); *n* (%); Mean ± SD.

*P*-values were corrected for multiple comparisons using the Holm-Bonferroni procedure.

GRS, global radial strain; GCS, global circumferential strain; GLS, global longitudinal strain; PSSR, peak systolic strain rate; PDSR, peak diastolic strain rate; εs, total strain; εe, passive strain; εa, active strain; SRs, peak positive strain rate; SRe, peak early negative strain rate; SRa, peak late negative strain rate.

* *P* < 0.05 vs. controls.

♯ *P* < 0.05 vs. HTN-only group.

LA deformation analysis revealed significant impairment in reservoir and conduit function across the control, HTN-only and HTN-T2DM groups (Figure 3). Compared to controls, both hypertensive groups had significantly lower εs, εe, SRs, and SRe (all *P* < 0.01). Importantly, the HTN-T2DM group exhibited further deterioration in reservoir (εs: *P* = 0.0175; SRs: *P* = 0.0337) and conduit (εe: *P* = 0.0011; SRe: *P* = 0.0364) function relative to the HTN-only group. In contrast, the pump function parameter (εa and SRa) was preserved among all groups (overall *P* > 0.05).

### 3.4 Correlation analysis of LACI and myocardial deformation

**Figure 5:**
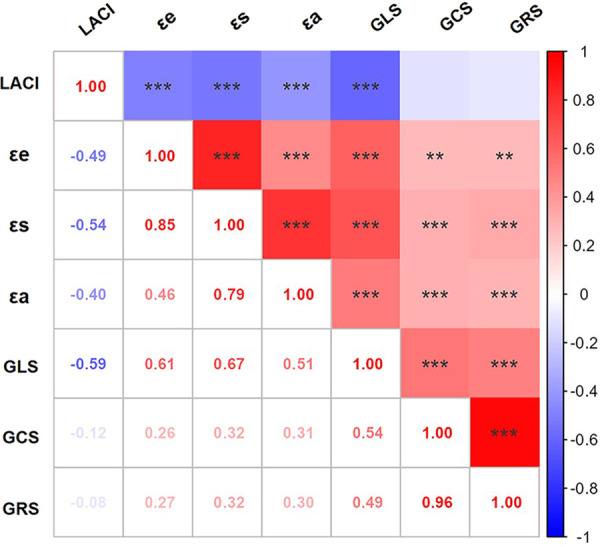
Interrelationships between LACI and left atrioventricular strain parameters in hypertensive patients. The heatmap color intensity reflects the correlation magnitude/direction (red: positive; blue: negative), with bivariate coefficients displayed numerically. *, *P* < 0.05; **, *P* < 0.01; ***, *P* < 0.001.

Spearman correlation analysis revealed significant associations among cardiac MRI-derived parameters, as summarized in the heatmap (Figure 5). Key findings include the following: LACI demonstrated moderate to strong negative correlations with all LA phasic strain parameters, particularly with reservoir strain (εs: *ρ* = −0.54, *P* < 0.001), conduit strain (εe: *ρ* = −0.49, *P* < 0.001), and peak global longitudinal strain of the left ventricle (GLS: *ρ* = −0.59, *P* < 0.001). In contrast, GLS showed positive correlations with LA deformation parameters, including reservoir strain (εs, *ρ* = 0.67, *P* < 0.001), conduit strain (εe, *ρ* = 0.61, *P* < 0.001), and active strain (εa: *ρ* = 0.51, *P* < 0.001).

### 3.5 Correlations between clinical metabolic indicators and cardiac parameters

Details are shown in Supplementary Table S1. To explore the relationship between metabolic dysregulation and cardiac alterations, Spearman correlation analyses were performed between key metabolic indicators and CMR-derived parameters. DM.duration showed prominent associations with cardiac structure and function: positively correlated with LV remodelling (Mi; *ρ* = 0.283, Ri; *ρ* = 0.354) and LACI (*ρ* = 0.372, all *P* < 0.001); negatively correlated with LV deformation (GLS; *ρ* = −0.399, εs; *ρ* = −0.396, εe; *ρ* = −0.448, all *P* < 0.001), indicating longer DM duration links to atrioventricular uncoupling and impaired myocardial deformation. HbA1c exhibited similar adverse patterns: positively associated with LVMi (*ρ* = 0.181, *P* = 0.018), Ri (*ρ* = 0.372) and LACI (*ρ* = 0.250, both *P* < 0.001); negatively correlated with GLS (*ρ* = −0.332), εs (*ρ* = −0.249) and εe (*ρ* = −0.313, all *P* < 0.001). FBG was positively correlated with LVRi (*ρ* = 0.197, *P* = 0.010) and negatively with GLS (*ρ* = −0.176, *P* = 0.021). Notably, HDL had protective effects: positively correlated with GLS (*ρ* = 0.249, *P* < 0.001), εs (*ρ* = 0.171, *P* = 0.025) and εe (*ρ* = 0.163, *P* = 0.033); negatively associated with LVRi (*ρ* = −0.265, *P* < 0.001) and LACI (*ρ* = −0.185, *P* = 0.015).

### 3.6 Multivariable regression analysis of cardiac function in overall and hypertensive cohorts

Multivariable linear regression models were employed to examine the independent associations between clinical variables and cardiac deformation parameters (εs, εe, GLS), and the left atrioventricular coupling index in both the overall study population and a subgroup of hypertensive patients.

In the model incorporating the entire cohort (Table 4), both HTN and HTN-T2DM were independently associated with significantly reduced left atrial reservoir function (εs), conduit function (εe), and reduced left ventricular GLS (all *P* < 0.05). The reduction in strain parameters was more pronounced in the HTN-T2DM group compared to the HTN-only group (e.g., εs: *β* = −11.96 vs. −5.67). Conversely, both HTN-only and HTN-T2DM were associated with a significant increase in LACI (*β* = 5.52 and 8.10, respectively; both *P* < 0.001), indicating impaired left atrioventricular coupling. Additionally, age and male sex were consistently associated with myocardial deformation and LACI.

**Table 4:** Multivariable linear regression analyses for left atrial and left ventricular strain parameters in the overall study population.

Characteristics	εs (Adjusted *R*^2^ = 0.241)			εe (Adjusted *R*^2^ = 0.376)			GLS (Adjusted *R*^2^ = 0.325)			LACI (Adjusted *R*^2^ = 0.264)		
	β	95% CI	*P*	β	95% CI	*P*-value	β	95% CI	*P*-value	β	95% CI	*P*-value
HTN	−5.67	−10.88, −0.46	0.034	−5.89	−9.50, −2.29	0.002	−2.27	−3.31, −1.23	<0.001	5.52	2.80, 8.24	<0.001
HTN-T2DM	−11.96	−17.85, −6.07	<0.001	−11.62	−15.69, −7.54	<0.001	−3.65	−4.83, −2.47	<0.001	8.10	5.02, 11.17	<0.001
Age	−0.39	−0.56, −0.21	<0.001	−0.41	−0.53, −0.29	<0.001	−0.03	−0.06, 0.01	0.124	0.19	0.10, 0.28	<0.001
Sex/male	−4.92	−8.81, −1.03	0.014	−3.60	−6.29, −0.90	0.010	−1.24	−2.02, −0.46	0.002	−0.37	−2.40, 1.66	0.722
BMI	−0.42	−0.92, 0.07	0.094	−0.13	−0.47, 0.21	0.458	−0.02	−0.11, 0.08	0.764	0.09	−0.17, 0.35	0.497
Heart rate	−0.04	−0.19, 0.10	0.554	−0.08	−0.18, 0.02	0.113	−0.02	−0.05, 0.01	0.275	0.01	−0.06, 0.09	0.747
SBP	−0.05	−0.15, 0.04	0.261	−0.03	−0.10, 0.03	0.325	−0.02	−0.04, 0.00	0.029	0.01	−0.04, 0.06	0.636
HDL-C	2.47	−4.51, 9.45	0.489	0.43	−4.40, 5.26	0.861	0.96	−0.44, 2.36	0.181	−3.52	−7.16, 0.12	0.060
LDL-C	0.03	−2.29, 2.35	0.982	−0.01	−1.62, 1.59	0.987	0.05	−0.41, 0.52	0.823	0.07	−1.14, 1.28	0.914
TyG	1.96	−1.08, 5.00	0.209	0.94	−1.16, 3.05	0.381	0.10	−0.51, 0.71	0.747	−1.89	−3.48, −0.30	0.021

CI, confidence interval, Other abbreviations are as defined in Tables 1–3.

Multivariable linear regression analyses within the hypertensive subgroup (Table 5) revealed that comorbid T2DM was independently associated with impaired left atrioventricular deformation parameters after adjusting for age, sex, BMI, heart rate, SBP, hypertension duration, HDL-C, LDL-C, and TyG. Specifically, T2DM was a significant independent predictor of reduced LA reservoir function (εs: *β* = −6.09, *P* = 0.018), impaired LA conduit function (εe: *β* = −5.58, *P* = 0.002), and worsened LV systolic function (GLS: *β* = −1.37, *P* = 0.010). The models explained 17.4%, 28.6%, and 14.4% of the variance in εs, εe, and GLS, respectively.

**Table 5:** Multivariable linear regression analyses of LA and LV strain in all hypertension patients.

Characteristics	εs (Adjusted *R*^2^ = 0.174)			εe (Adjusted *R*^2^ = 0.286)			GLS (Adjusted *R*^2^ = 0.144)			LACI (Adjusted *R*^2^ = 0.124)		
	β	95% CI	*P*	β	95% CI	*P*-value	β	95% CI	*P*-value	β	95% CI	*P*-value
T2DM	−6.09	−11.06, −1.13	0.018	−5.58	−8.98, −2.18	0.002	−1.37	−2.40, −0.34	0.010	2.09	−0.61, 4.79	0.131
Age	−0.37	−0.59, −0.15	0.001	−0.41	−0.57, −0.26	<0.001	−0.03	−0.07, 0.02	0.281	0.24	0.12, 0.36	<0.001
Sex/male	−5.57	−10.37, −0.78	0.025	−3.79	−7.08, −0.50	0.026	−1.49	−2.49, −0.50	0.004	0.11	−2.51, 2.72	0.937
BMI	−0.40	−1.00, 0.21	0.200	−0.08	−0.50, 0.33	0.697	−0.02	−0.15, 0.10	0.710	0.11	−0.22, 0.44	0.506
Heart rate	−0.07	−0.25, 0.11	0.426	−0.13	−0.26, −0.01	0.035	−0.01	−0.05, 0.02	0.496	0.01	−0.09, 0.11	0.828
SBP	−0.07	−0.17, 0.04	0.226	−0.05	−0.12, 0.03	0.216	−0.02	−0.05, 0.00	0.034	0.03	−0.03, 0.08	0.396
HTN duration	−0.04	−0.54, 0.46	0.867	0.10	−0.24, 0.44	0.561	−0.04	−0.14, 0.06	0.444	−0.29	−0.56, −0.01	0.041
HDL-C	6.04	−2.34, 14.41	0.160	2.41	−3.33, 8.15	0.413	1.18	−0.56, 2.92	0.186	−3.68	−8.24, 0.88	0.116
LDL-C	0.52	−2.26, 3.29	0.716	0.37	−1.53, 2.27	0.703	0.06	−0.51, 0.64	0.826	−0.32	−1.83, 1.19	0.681
TyG	2.25	−1.43, 5.93	0.233	1.13	−1.40, 3.65	0.383	0.09	−0.67, 0.86	0.816	−1.69	−3.69, 0.31	0.101

### 3.7 Reproducibility

Reproducibility analysis confirmed the robustness of CMR-FT measurements across all deformation parameters (Supplementary Table S2). Intraobserver ICCs ranged from 0.92 (GCS) to 0.98 (εs), while interobserver ICCs varied between 0.87 (GCS) and 0.97 (εs).

## 4 Discussion

This study utilised 3.0 T CMR feature-tracking to investigate the additional impact of T2DM on cardiac deformation and atrioventricular coupling in hypertensive patients with preserved ejection fraction. The principal findings are as follows: [1] Graded cardiac impairment was observed across groups, evident as worsening LV remodelling, LA reservoir/conduit dysfunction and reduced GLS from controls to HTN-only and HTN-T2DM patients. [2] Critically, multivariable regression identified comorbid T2DM as an independent factor of impaired LA reservoir/conduit function and reduced LV deformation, indicating an additional detrimental effect beyond hypertension alone. [3] Diabetes duration and HbA1c correlated with greater cardiac remodelling, elevated LACI and reduced myocardial deformation. [4] Notably, LACI, significantly elevated in the HTN-T2DM group, represents an integrated marker of the atrioventricular uncoupling imposed by the dual burden of hemodynamic and metabolic stress.

Collectively, these observations indicate that in early hypertensive heart disease, concomitant T2DM is associated with more severe subclinical cardiac dysfunction and atrioventricular uncoupling. This study provides imaging-based evidence supporting early detection and integrated management of hemodynamic and metabolic risk factors in this high-risk population.

Our data reveal a distinct pattern of early cardiac involvement in hypertensive heart disease exacerbated by T2DM. While LV ejection fraction remained normal, LV deformation (GLS) showed progressive impairment, a marker sensitive to subendocardial dysfunction often linked to microvascular disease in diabetes (19, 20). More strikingly, LA phasic function demonstrated a selective vulnerability: reservoir (εs) and conduit (εe) strains were significantly and incrementally reduced across groups, whereas the booster pump function (εa) was preserved.

This pattern diverges from that reported by Shi et al. (11), who found LA pump dysfunction (εa) to be prominently affected in a diabetic cohort with superimposed hypertension. This discrepancy is mechanistically informative. In our hypertension-first cohort, the initial hemodynamic insult may induce compensatory LA remodeling that temporarily preserves late-diastolic contraction (21). The subsequent metabolic insult of T2DM, characterized by inflammation, fibrosis, and reduced atrial compliance (22, 23), then preferentially disrupts the earlier, more compliance-dependent phases of LA function (reservoir and conduit), which are directly exposed to rising LV filling pressures (24). Critically, both patterns of LA dysfunction, whether affecting early or late diastolic phases, converge on a common pathological substrate: the development of interstitial atrial fibrosis, which is a well-established pro-arrhythmic substrate that elevates the risk of adverse cardiovascular outcomes, including atrial fibrillation (AF) and heart failure (25). This sequence suggests that the “first hit” (hypertension vs. T2DM) may dictate the initial phenotypic expression of LA dysfunction, a hypothesis requiring longitudinal validation.

The pathophysiological interplay described above culminates in a measurable state of mechanical discoordination between the LA and LV. The left atrioventricular coupling index, defined as the ratio of LA minimal volume to LV end-diastolic volume (18, 26), quantifies this relationship. Our finding that LACI was most elevated in the HTN-T2DM group is a central result. It signifies that for a given LV filling volume, the atrium must operate at a larger minimal size, a direct reflection of elevated filling pressures and reduced atrial compliance.

Importantly, LACI did not operate in isolation. It exhibited strong negative correlations with both ventricular (GLS) and atrial (εs, εe) deformation parameters. This triangulation of data confirms that LACI is not merely a volumetric ratio but a sensitive integrator of the combined mechanical burden from hypertension (pressure overload) and T2DM (metabolic stiffening). Its correlation with diabetes duration and HbA1c further underscores its role as a potential imaging biomarker of cumulative cardiometabolic risk. This aligns with and extends a growing body of evidence that has established the prognostic value of elevated LACI for predicting major adverse cardiac events, including incident heart failure and atrial fibrillation (18, 26–28). Our study demonstrates that this prognostic marker is associated with the very early, additive uncoupling in patients with hypertension and T2DM, which highlights an association between subclinical pathology detected by CMR and known pathways of adverse cardiovascular outcomes.

The clinical significance of our study lies in its ability to identify a high-risk cardiac phenotype among asymptomatic individuals. The impairments we documented occurred in the absence of heart failure symptoms or significant structural remodelling, highlighting a preclinical window for intervention. This clear dissociation between declining mechanical function and preserved structure underscores that dysfunction precedes overt structural abnormalities (29–32).

Firstly, our findings provide compelling imaging-based evidence for the necessity of integrated cardiometabolic management. The independent and additive effect of T2DM on atrioventricular mechanics, even after adjusting for blood pressure, indicates that managing hypertension alone is likely insufficient for this population. Therapeutic strategies must concurrently address the metabolic derangements of diabetes to mitigate the progression of subclinical dysfunction (33, 34).

Secondly, our study highlights the potential of CMR-derived advanced indices for refined risk stratification. Parameters such as LACI and LA conduit/reservoir strain offer a quantitative and sensitive assessment of the integrated atrioventricular burden imposed by coexisting hypertension and T2DM. Elevated LACI, in particular, serves as an integrative biomarker that encapsulates the hemodynamic and metabolic stress leading to mechanical uncoupling, a state strongly linked to future adverse cardiovascular outcomes including heart failure and atrial fibrillation (25, 28, 35, 36). By quantifying this uncoupling, these metrics could help identify hypertensive patients with T2DM who, despite a preserved EF, are on a steeper trajectory toward heart failure with preserved EF (HFpEF), a condition with similar features of diastolic dysfunction and atrial myopathy (4, 5).

Therefore, incorporating LACI and LA strain into routine CMR assessment could enhance early risk stratification, identifying a subgroup that warrants more intensive monitoring and aggressive risk factor modification. Future prospective studies are needed to validate these indices as tools for guiding personalized primary prevention strategies. The ultimate goal is to move beyond a one-size-fits-all approach and use such detailed phenotyping to tailor the intensity and type of lifestyle or pharmacological interventions, aiming to prevent the transition from subclinical atrioventricular uncoupling to overt cardiovascular events.

## 5 Limitations

This study has limitations. First, its cross-sectional design precludes causal inference regarding the additive effect of T2DM. Second, generalizability may be limited by the single-center design, modest sample size, and the use of a symptomatic control group, which also precluded a formal analysis of medication effects. Finally, despite statistical adjustments, residual confounding from unmeasured factors (e.g., detailed treatment regimens) cannot be excluded.

## 6 Conclusions

In conclusion, hypertensive patients with concomitant type 2 diabetes mellitus exhibit more pronounced left ventricular remodelling, impaired left atrial reservoir and conduit deformation, and worse left atrioventricular uncoupling compared to those with hypertension alone. These adverse changes were most strongly associated with longer diabetes duration and poorer glycaemic control. The findings underscore the importance of integrated cardiometabolic management and highlight the potential utility of advanced cardiac magnetic resonance indices in the early identification of subclinical cardiac dysfunction in this high-risk population.

## Data Availability

The raw data supporting the conclusions of this article will be made available by the authors, without undue reservation.
