# Supplementary material for: Association of type 2 diabetes with left atrioventricular coupling and myocardial deformation in hypertension: a 3.0 T cardiac magnetic resonance study
Source: Front Cardiovasc Med. 2026 Feb 26;13:1753368. doi: 10.3389/fcvm.2026.1753368 (PMC12979156; doi:10.3389/fcvm.2026.1753368)
Supplement: Supplementary file 1 [file Supplementaryfile1.docx]

Supplementary Table 1 Correlations between Clinical Metabolic Indicators and Cardiac Parameters

|  | LVMI | LVRI | GLPS | εs | εe | LACI |
| --- | --- | --- | --- | --- | --- | --- |
|  | ρ | ρ | ρ | ρ | ρ | ρ |
| Age | 0.107 | 0.203** | -0.133 | -0.305*** | -0.432*** | 0.318*** |
| Sex | 0.258*** | 0.232*** | -0.231*** | -0.157* | -0.138 | -0.022 |
| BMI | 0.040 | 0.157* | -0.138 | -0.172* | -0.116 | 0.088 |
| Heart rate | -0.053 | 0.001 | -0.039 | 0.016 | -0.018 | -0.072 |
| SBP | 0.357*** | 0.372*** | -0.331*** | -0.247** | -0.265*** | 0.133 |
| HTN duration | 0.401*** | 0.447*** | -0.471*** | -0.403*** | -0.443*** | 0.342*** |
| T2DM duration | 0.283*** | 0.354*** | -0.399*** | -0.396*** | -0.448*** | 0.372*** |
| HbA1c | 0.181* | 0.372*** | -0.332*** | -0.249*** | -0.313*** | 0.250*** |
| FBG | 0.066 | 0.197** | -0.176* | -0.120 | -0.146 | 0.133 |
| TC | -0.102 | -0.075 | 0.058 | 0.064 | 0.058 | -0.083 |
| TG | 0.059 | 0.063 | -0.084 | 0.075 | 0.022 | -0.079 |
| HDL-C | -0.237** | -0.265*** | 0.249*** | 0.171* | 0.163* | -0.185* |
| LDL-C | -0.130 | -0.093 | 0.076 | 0.071 | 0.092 | -0.050 |
| TyG | 0.083 | 0.121 | -0.133 | 0.025 | -0.024 | 0.009 |
| Note: ρ denotes Spearman's rank correlation coefficient.  Statistical significance is indicated as follows: * *P* < 0.05, ** *P* < 0.01, *** *P* < 0.001. | | | | | | |
