# Supplementary material for: Association of type 2 diabetes with left atrioventricular coupling and myocardial deformation in hypertension: a 3.0 T cardiac magnetic resonance study
Source: Front Cardiovasc Med. 2026 Feb 26;13:1753368. doi: 10.3389/fcvm.2026.1753368 (PMC12979156; doi:10.3389/fcvm.2026.1753368)
Supplement: Supplementary file 2 [file Supplementaryfile2.docx]

Supplementary Table 2 Intra- and inter-observer reproducibility of CMR-FT derived LV and LA strain parameters

|  | Interobserver | | Intraobserver | |
| --- | --- | --- | --- | --- |
|  | ICC | 95%CI | ICC | 95%CI |
| εs (%) | 0.97 | 0.94 - 0.98 | 0.98 | 0.95 - 0.99 |
| εe (%) | 0.96 | 0.93 - 0.98 | 0.97 | 0.95 - 0.98 |
| εa (%) | 0.88 | 0.78 - 0.93 | 0.94 | 0.88 - 0.96 |
| GLS (%) | 0.91 | 0.85 - 0.95 | 0.95 | 0.91 - 0.97 |
| GRS (%) | 0.95 | 0.91 - 0.97 | 0.96 | 0.93 - 0.98 |
| GCS (%) | 0.87 | 0.77 - 0.93 | 0.92 | 0.86 - 0.95 |
| Abbreviations: ICC, intraclass correlation coefficient; ICC, intraclass correlation coefficient; Other abbreviations are as defined in Tables 2 | | | | |
